# Supplementary material for: Golem: an algorithm for robust experiment and process optimization
Source: Chem Sci. 2021 Oct 12;12(44):14792–807. doi: 10.1039/d1sc01545a (PMC8597856; doi:10.1039/d1sc01545a)
Supplement: SC-012-D1SC01545A-s001 [file SC-012-D1SC01545A-s001.pdf]

Electronic Supplementary Material (ESI) for Chemical Science.
This journal is © The Royal Society of Chemistry 2021

Supplementary Information

Golem: An algorithm for robust experiment and process optimization

Matteo Aldeghi,[1,2,3] Florian Häse,[4,1,2,3] Riley J. Hickman,[2,3] Isaac Tamblyn,[1,5] Alán Aspuru-Guzik[1,2,3,6]

[1] *Vector Institute for Artificial Intelligence, Toronto, ON, Canada*
[2] *Chemical Physics Theory Group, Department of Chemistry, University of Toronto, Toronto, ON, Canada*
[3] *Department of Computer Science, University of Toronto, Toronto, ON, Canada*
[4] *Department of Chemistry and Chemical Biology, Harvard University, Cambridge, MA, USA*
[5] *National Research Council of Canada, Ottawa, ON, Canada*
[6] *Lebovic Fellow, Canadian Institute for Advanced Research, Toronto, ON, Canada*

S.1. FORMULATING GOLEM

Consider a sequential optimization in which the goal is to find a set of input conditions $\boldsymbol{x} \in \mathcal{X}$ corresponding to the global minimum of the function $f : \mathcal{X} \mapsto \mathbb{R}$,

$$\boldsymbol{x}^* = \underset{\boldsymbol{x}\in\mathcal{X}}{\operatorname{argmin}}\, f(\boldsymbol{x}). \tag{5}$$

At each iteration k, we query parameter values $\boldsymbol{x}_k \in \mathcal{X}$, where $\mathcal{X}$ is a compact subset of Euclidean space $\mathbb{R}^D$ and $D \in \mathbb{N}^*$. However, while the desired query location $\boldsymbol{x}$ can be precisely controlled, uncertainty in the execution results in $\widetilde{\boldsymbol{x}} = \boldsymbol{x} + \boldsymbol{\delta}$ being the input realized, where $\boldsymbol{\delta}$ is a random variable with probability density $p(\boldsymbol{\delta}|\boldsymbol{x})$. Thus, after K optimization iterations, we will have built a dataset $\widetilde{\mathcal{D}}_K = \{\boldsymbol{x}_k, f(\widetilde{\boldsymbol{x}}_k)\}_{k=1}^K$, where uncertainty in the inputs causes stochastic evaluations of the response function $f(\boldsymbol{x})$. A noiseless dataset $\mathcal{D}_K = \{\boldsymbol{x}_k, f(\boldsymbol{x}_k)\}_{k=1}^K$ can be obtained when $p(\boldsymbol{\delta}|\boldsymbol{x})$ is a delta function.

We seek a robustness measure $g(\boldsymbol{x}_k)$ that will allow us to estimate the merit of each solution $\boldsymbol{x}_k$ given its uncertainty. This would not only allow for *post-hoc* rescaling of the merit of each parameter location based on its robustness, but also to direct experiment planning algorithms towards robust optima during the optimization campaign.

A natural choice is to take the expectation of $f(\widetilde{\boldsymbol{x}}_k)$ by marginalizing over the input uncertainty, such that $g(\boldsymbol{x}_k) = \mathbb{E}[f(\widetilde{\boldsymbol{x}}_k)]$. Consider $\boldsymbol{x}_k$ to be the query location at iteration k, where we would like to evaluate $f(\boldsymbol{x}_k)$. The location actually realized, due to the uncertainty, is $\widetilde{\boldsymbol{x}}_k = \boldsymbol{x}_k + \boldsymbol{\delta}$, where $\boldsymbol{\delta}$ is a random variable with probability density $p(\boldsymbol{\delta}|\boldsymbol{x}_k)$. In principle, the expectation of $f(\widetilde{\boldsymbol{x}}_k)$ given $p(\widetilde{\boldsymbol{x}}_k)$ can be obtained as follows:

$$\mathbb{E}[f(\widetilde{\boldsymbol{x}}_k)] = \int_{\mathbb{R}^D} f(\boldsymbol{x}) p(\widetilde{\boldsymbol{x}}_k) d\boldsymbol{x} \tag{6}$$

$$= \int_{\mathbb{R}^D} f(\boldsymbol{x}) p(\boldsymbol{x}_k + \boldsymbol{\delta}) d\boldsymbol{x}, \tag{7}$$

with integration over the support of $p(\widetilde{\boldsymbol{x}}_k)$, because $p(\boldsymbol{\delta}|\boldsymbol{x}_k)$ may extend beyond the bounds of $\mathcal{X}$. Here we assume integration over $\mathbb{R}^D$ without loss of generality. If physical bounds are present, this can be reflected in the support of $p(\boldsymbol{\delta}|\boldsymbol{x}_k)$; for instance, the uncertainty in volume of dispensed liquid may be modelled by a gamma distribution, given that volume dispensed can only be equal to or greater than zero. Eq. 6 is another way to write the expectation obtained by marginalising over the noise factors $\boldsymbol{\delta}$,

$$\mathbb{E}[f(\widetilde{\boldsymbol{x}}_k)] = \int_{\mathbb{R}^D} f(\boldsymbol{x}_k + \boldsymbol{\delta}) p(\boldsymbol{\delta}) d\boldsymbol{\delta}. \tag{8}$$

More explicitly,

$$\mathbb{E}[f(\widetilde{\boldsymbol{x}}_k)] = \int_{\mathbb{R}^D} f(\boldsymbol{x}_k + \boldsymbol{t}) p_{\boldsymbol{\delta}}(\boldsymbol{t}) d\boldsymbol{t}. \tag{9}$$

Defining $\boldsymbol{x} = \boldsymbol{x}_k + \boldsymbol{t}$, $d\boldsymbol{x} = d\boldsymbol{t}$, and given that $p_{\boldsymbol{\delta}}(\boldsymbol{x} - \boldsymbol{x}_k) = p_{\boldsymbol{x}_k+\boldsymbol{\delta}}(\boldsymbol{x})$,

$$\mathbb{E}[f(\widetilde{\boldsymbol{x}}_k)] = \int_{\mathbb{R}^D} f(\boldsymbol{x}) p_{\boldsymbol{\delta}}(\boldsymbol{x} - \boldsymbol{x}_k) d\boldsymbol{x} \tag{10}$$

$$= \int_{\mathbb{R}^D} f(\boldsymbol{x}) p_{\boldsymbol{x}_k+\boldsymbol{\delta}}(\boldsymbol{x}) d\boldsymbol{x}. \tag{11}$$

Eq. 6 as shown above is recovered when simplifying the notation with $p_{\boldsymbol{x}_k+\boldsymbol{\delta}}(\boldsymbol{x}) = p(\boldsymbol{x}_k + \boldsymbol{\delta})$. Finally, note that other integrated measures of robustness can be defined, for instance considering higher moments of the objective function.[1,2]

While $f(\boldsymbol{x})$ is unknown, an approximation $\hat{f}(\boldsymbol{x})$ can be built from $\mathcal{D}_K$ or $\widetilde{\mathcal{D}}_K$. For simplicity, we assume $\hat{f}(\boldsymbol{x}) \approx f(\boldsymbol{x})$ and from now on will refer to $\hat{f}(\boldsymbol{x})$ simply as $f(\boldsymbol{x})$. If we can solve the above integral efficiently, we can then use $\mathbb{E}[f(\widetilde{\boldsymbol{x}}_k)]$ as the robust merit $g(\boldsymbol{x})$ for each condition $\boldsymbol{x}_k$, and $\mathcal{G}_K = \{\boldsymbol{x}_k, g_k\}_{k=1}^K$ could be used with an experiment planning algorithm of choice to solve the robust optimization problem as

$$\boldsymbol{x}^* = \underset{\boldsymbol{x} \in \mathcal{X}}{\operatorname{argmin}}\, g(\boldsymbol{x}). \tag{12}$$

However, there is no closed form solution of Eq. 6 for most combinations of $f(\boldsymbol{x})$ and $p(\widetilde{\boldsymbol{x}}_k)$. Its numerical approximation is expensive, becoming intractable with increasing dimensionality and number of samples.

A. Continuous input variables

The space over which $p(\widetilde{\boldsymbol{x}})$ is supported (here considered to be $\mathbb{R}^D \supset \mathcal{X}$) can be partitioned into $M \in \mathbb{N}^*$ non-overlapping tiles $\{\mathcal{T}_m^D\}_{m=1}^M$, with $\mathcal{T}_m^D \subset \mathbb{R}^D \; \forall \, m \leq M$, to create a D-dimensional tessellation. With this discretization, Eq. 6 can be decomposed into a finite series with integration over each tile $\mathcal{T}_m^D$:

$$\mathbb{E}[f(\widetilde{\boldsymbol{x}}_k)] = \sum_{m=1}^{M} \int_{\mathcal{T}_m^D} f(\boldsymbol{x}) p(\widetilde{\boldsymbol{x}}_k) d\boldsymbol{x}. \tag{13}$$

Assuming a piecewise constant model of $f(\boldsymbol{x})$, such as a regression tree, $f(\boldsymbol{x})$ is constant within the partition $\mathcal{T}_m^D$ and can be brought outside the integral:

$$\mathbb{E}[f(\widetilde{\boldsymbol{x}}_k)] = \sum_{m=1}^{M} f_m \int_{\mathcal{T}_m^D} p(\widetilde{\boldsymbol{x}}_k) d\boldsymbol{x}. \tag{14}$$

The integral over $\mathcal{T}_m^D$

$$\int_{\mathcal{T}_m^D} p(\widetilde{\boldsymbol{x}}_k) d\boldsymbol{x} = P(\widetilde{\boldsymbol{x}}_k \in \mathcal{T}_m^D) \tag{15}$$

is the probability of $\boldsymbol{x}_k$ being in tile m, given the uncertainty $p(\boldsymbol{\delta}_k)$. Therefore, $\mathbb{E}[f(\widetilde{\boldsymbol{x}}_k)]$ is effectively a weighted average,

$$\mathbb{E}[f(\widetilde{\boldsymbol{x}}_k)] = \sum_{m=1}^{M} f_m \cdot P(\widetilde{\boldsymbol{x}}_k \in \mathcal{T}_m^D), \tag{16}$$

where all possible outcomes are weighted by their probability given the targeted parameter location $\boldsymbol{x}_k$. Assuming independent input uncertainties, $P(\widetilde{\boldsymbol{x}}_k \in \mathcal{T}_m^D)$ can be factorized:

$$P(\widetilde{\boldsymbol{x}}_k \in \mathcal{T}_m^D) = \prod_{d=1}^{D} P(\widetilde{x}_{k,d} \in \mathcal{T}_{m,d}). \tag{17}$$

The probability $P(\widetilde{x}_{k,d} \in \mathcal{T}_{m,d})$ is obtained from the cumulative distribution function $F_{k,d}$ of $p(\widetilde{\boldsymbol{x}}_k)$, evaluated at the upper and lower bounds of tile m in dimension d,

$$P(\widetilde{x}_{k,d} \in \mathcal{T}_{m,d}) = F_{k,d}(\max_d \ \mathcal{T}_m^D) - F_{k,d}(\min_d \ \mathcal{T}_m^D), \tag{18}$$

where $\max_d \ \mathcal{T}_m^D$ and $\min_d \ \mathcal{T}_m^D$ are the upper and lower bounds of tile m, respectively, in the d dimension. $F_{k,d}(a) = \int_{-\infty}^{a} p(\widetilde{x}_{k,d}) dx_d$ is the cumulative distribution function of $p(\widetilde{x}_k)$ in the d dimension. It thus follows that, combining Eq. 14-18, for a piecewise constant model $f(\boldsymbol{x})$ and any parametric distribution $p(\widetilde{\boldsymbol{x}}_k)$ with known F_k, the desired expectation can be computed as

$$\mathbb{E}[f(\widetilde{\boldsymbol{x}}_k)] = \sum_{m=1}^{M} f_m \prod_{d=1}^{D} [F_{k,d}(\max_d \ \mathcal{T}_m^D) - F_{k,d}(\min_d \ \mathcal{T}_m^D)]. \tag{19}$$

In this work, we model $f(\boldsymbol{x})$ with single regression trees as well as their ensemble variants, like random forest and extremely randomized trees.[3,4] When ensembles are used, a different expectation $\mathbb{E}[f_t(\widetilde{\boldsymbol{x}}_k)]$ is obtained for each tree t, in which case we take their average as the most reliable estimate of robustness:

$$\mathbb{E}[f(\widetilde{\boldsymbol{x}}_k)] = \frac{1}{T} \sum_{t=1}^{T} \sum_{m=1}^{M} f_{t,m} \prod_{d=1}^{D} [F_{k,d}(\max_d \ \mathcal{T}_{t,m}^D) - F_{k,d}(\min_d \ \mathcal{T}_{t,m}^D)]. \tag{20}$$

B. Discrete input variables

Tree-based machine learning approaches can also take discrete and categorical variables as features, such that uncertainty in these types of inputs can also be handled by Golem. When optimizing over a discrete space $\mathcal{X} \subset \mathbb{N}^D$, both $\boldsymbol{x} \in \mathcal{X}$ and $\boldsymbol{\delta} \in \mathbb{N}^D$ are discrete and the expectation of f is expressed as a sum over $\mathbb{N}^D$,

$$\mathbb{E}[f(\widetilde{\boldsymbol{x}}_k)] = \sum_{\mathbb{N}^D} f(\boldsymbol{x}) p(\widetilde{\boldsymbol{x}}_k), \tag{21}$$

where $p(\widetilde{\boldsymbol{x}}_k)$ is a discrete probability distribution. The $\mathbb{N}^D$ space supporting this distribution can be partitioned into $M \in \mathbb{N}^*$ non-overlapping tiles $\{\mathcal{T}_m^D\}_{m=1}^M$, with $\mathcal{T}_m^D \subset \mathbb{N}^D \ \forall \ m \leq M$,

$$\mathbb{E}[f(\widetilde{\boldsymbol{x}}_k)] = \sum_{m=1}^{M} \sum_{\mathcal{T}_m^D} f(\boldsymbol{x}) p(\widetilde{\boldsymbol{x}}_k). \tag{22}$$

For a model that takes a constant f_m value within each tile $\mathcal{T}_m^D$,

$$\mathbb{E}[f(\widetilde{\boldsymbol{x}}_k)] = \sum_{m=1}^{M} \Big(f_m \sum_{\mathcal{T}_m^D} p(\widetilde{\boldsymbol{x}}_k) \Big) \tag{23}$$

$$= \sum_{m=1}^{M} f_m \cdot P(\widetilde{\boldsymbol{x}}_k \in \mathcal{T}_m^D). \tag{24}$$

As for continuous variables, we assume independent uncertainty across input variables, such that $P(\widetilde{\boldsymbol{x}}_k \in \mathcal{T}_m^D)$ factorises. The rest of the derivation then follows the same argument as for continuous variables. Thus, the only difference to continuous variables is that $\widetilde{\boldsymbol{x}}$ and its probability distributions are discrete.

C. Categorical input variables

If the optimization occurs over a D-dimensional space with $C \in \mathbb{N}^*$ categorical options, at each iteration we query a point $\boldsymbol{x}_k \in \mathbb{S}^{D\times C}$ that selects a category $\boldsymbol{z}_d$ for each dimension d. This information can be encoded as C-dimensional one-hot encoded vectors, $\mathbb{S}^{D\times C} = \{\boldsymbol{z} \in \mathbb{R}^{D\times C} | z_{d,c} \in \{0,1\}; \sum_{c=1}^{C} z_{d,c} = 1 \; \forall \; d \leq D\}$. The uncertainty over categorical variables can then be represented by any suitable probability distribution on the simplex, $p(\widetilde{\boldsymbol{x}}_k) \in \Delta^{D\times(C-1)} = \{p(\boldsymbol{z}) \in \mathbb{R}^{D\times C} | p(z_{d,c}) \in [0,1]; \sum_{c=1}^{C} p(z_{d,c}) = 1 \; \forall \; d \leq D\}$. In this scenario, the expectation of f queried at location $\boldsymbol{x}_k$ and considering the uncertainty due to $\boldsymbol{\delta}_k$ is

$$\mathbb{E}[f(\widetilde{\boldsymbol{x}}_k)] = \sum_{\mathbb{S}^{D\times C}} f(\boldsymbol{x}) p(\widetilde{\boldsymbol{x}}_k). \tag{25}$$

Similar to what was done before, we partition the space $\mathbb{S}^{D\times C}$ in $M \in \mathbb{N}^*$ non-overlapping tiles $\{\mathcal{T}_m^{D\times C}\}_{m=1}^{M}$, with $\mathcal{T}_m^{D\times C} \subset \mathbb{S}^{D\times C} \; \forall \; m \leq M$:

$$\mathbb{E}[f(\widetilde{\boldsymbol{x}}_k)] = \sum_{m=1}^{M} \sum_{\mathcal{T}_m^{D\times C}} f(\boldsymbol{x}) p(\widetilde{\boldsymbol{x}}_k). \tag{26}$$

For a model that assumes a constant f_m values within each tile $\mathcal{T}_m^{D\times C}$,

$$\mathbb{E}[f(\widetilde{\boldsymbol{x}}_k)] = \sum_{m=1}^{M} \Big(f_m \sum_{\mathcal{T}_m^{D\times C}} p(\widetilde{\boldsymbol{x}}_k) \Big) \tag{27}$$

$$= \sum_{m=1}^{M} f_m \cdot P(\widetilde{\boldsymbol{x}}_k \in \mathcal{T}_m^{D\times C}). \tag{28}$$

Assuming independent uncertainty across input variables,

$$P(\widetilde{\boldsymbol{x}}_k \in \mathcal{T}_m^{D\times C}) = \prod_{d=1}^{D} P(\widetilde{\boldsymbol{x}}_{k,d} \in \mathcal{T}_{m,d}^{1\times C}). \tag{29}$$

The probability of $\widetilde{\boldsymbol{x}}_{k,d}$ being in the $1 \times C$ dimensional tile $\mathcal{T}_{m,d}^{1\times C}$ can be readily computed from the user-defined probabilities $p(z_{d,c})$ indicating the uncertainty over categorical variables, such that

$$P(\widetilde{\boldsymbol{x}}_{k,d} \in \mathcal{T}_{m,d}^{1\times C}) = \sum_{c=1}^{C} p(z_{d,c}) \cdot \mathbb{I}(\boldsymbol{z}_d \in \mathcal{T}_{m,d}^{1\times C}), \tag{30}$$

where $\mathbb{I}$ is the indicator function, taking the value of 1 if the category $\boldsymbol{z}_d$ is in tile $\mathcal{T}_{m,d}^{1\times C}$ and 0 otherwise. In our implementation, we build trees until leaves are pure, which means that each tile $\mathcal{T}_{m,d}^{1\times C}$ will contain a single category (when at least one sample per category is present). However, this does not necessarily need to be the case and one might decide to limit tree depth for computational efficiency.

D. Multi-objective optimization

In addition to computing the expectation of $f(\boldsymbol{x})$, one can also consider its variance. As lower variance favors reproducibility, one might be interested in minimizing both $\mathbb{E}[f(\widetilde{\boldsymbol{x}}_k)]$ and $\sigma[f(\widetilde{\boldsymbol{x}}_k)] = Var[f(\widetilde{\boldsymbol{x}}_k)]^{\frac{1}{2}}$. The variance can easily be obtained as $Var[f(\widetilde{\boldsymbol{x}}_k)] = \mathbb{E}[f(\widetilde{\boldsymbol{x}}_k)^2] - \mathbb{E}[f(\widetilde{\boldsymbol{x}}_k)]^2$. With both $\mathbb{E}[f(\widetilde{\boldsymbol{x}}_k)]$ and $\sigma[f(\widetilde{\boldsymbol{x}}_k)]$ available, one can carry out a multi-objective optimization by building a robust merit $g(\boldsymbol{x})$ that takes both objectives into account, using any scalarizing function of choice. Figure S1 shows an example where a robust merit function is built via a weighted sum.

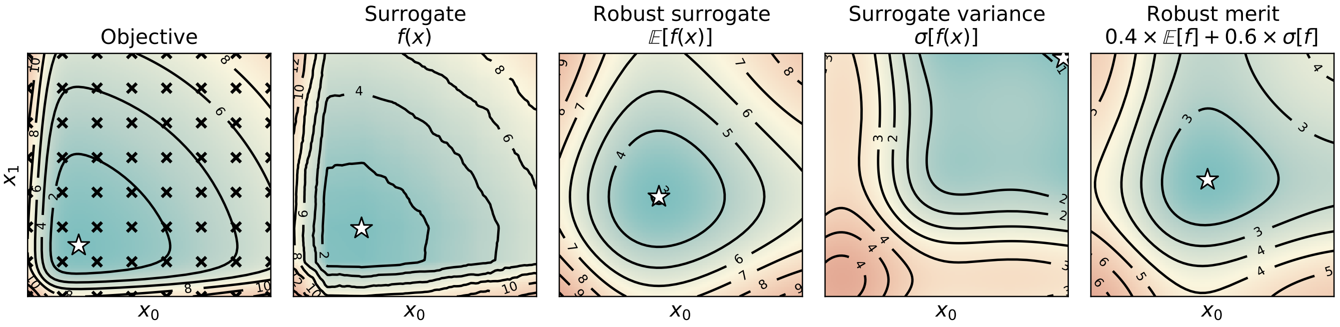

FIG. S1. Multi-objective optimization with Golem. The first plot on the left shows the *Cliff* objective function (section S.2.A). Golem's surrogate model was built using the 64 samples marked as black crosses. The model, in this case, was a forest of 100 extremely randomized trees[4]. The robust surrogate was built assuming normally distributed input noise, in both dimensions, and with unit standard deviation. The variability of the objective function under this noise model was computed as $\sigma[f(\tilde{\boldsymbol{x}})] = (\mathbb{E}[f(\tilde{\boldsymbol{x}})^2] - \mathbb{E}[f(\tilde{\boldsymbol{x}})]^2))^{\frac{1}{2}}$. The two objectives were combined into a single function to be optimized via the weighted sum $g(\boldsymbol{x}) = 0.4 \times \mathbb{E}[f(\tilde{\boldsymbol{x}})] + 0.6 \times \sigma[f(\tilde{\boldsymbol{x}})]$, where 0.4 and 0.6 are user-defined coefficients.

E. Golem's assumptions

Golem relies on three fundamental assumptions for its derivation as well as successful deployment. First, it is assumed that a piece-wise constant model, such as those obtained with tree-based algorithms, is able to provide an accurate surrogate model of the underlying objective function $f(\boldsymbol{x})$. In addition, it is assumed that such a surrogate model can be built from finite datasets $\mathcal{D}_K$ or $\widetilde{\mathcal{D}}_K$. It is expected that building an accurate surrogate model will be more challenging when using a noisy dataset $\widetilde{\mathcal{D}}_K$ based on stochastic queries of input conditions, than when using a noiseless dataset $\mathcal{D}_K$ based on deterministic ones. Second, it is assumed that the user knows and is able to accurately model input uncertainty via a parametric probability distribution $p(\boldsymbol{x})$. Finally, a necessary assumption in the above Golem's derivation is that the uncertainties of different input conditions are independent, such that, for instance, $p(x_i, x_j) = p(x_i)p(x_j)$ where i and j are two different input dimensions. This assumption might not always be satisfied depending on the input conditions and experimental setup. For instance, imagine that some uncertainty is associated with both target temperature and dispensed volume of liquid for a hypothetical experiment. If the liquid is first dispensed at room temperature and then heated to the desired target temperature, the errors in volume dispensed and target temperature are indeed likely independent. However, if the liquid is heated to a target temperature before dispensing, the (unknown, realized) temperature might affect viscosity, which in turn will have an effect on dispensing errors. That said, Golem allows for the uncertainty in one input variable to depend on the query location of all input variables, such that one can define $p(\tilde{x}_i \mid x_i, x_j)$. Using the same example from above, it is thus possible to specify how the dispensing uncertainty depends upon the target temperature, though not the realized, unknown one.

F. Computational scaling

To obtain the estimate of the robust merit for an input location $\boldsymbol{x}$, Golem evaluates Eq. 20 after having fitted the tree-based surrogate model. For S input locations, this involves performing operations over all input dimensions D, number of tiles M, and number of trees T. The time complexity of the algorithm (given an already trained tree-based model) thus scales linearly with respect to all these variables, $\mathcal{O}(S \times T \times M \times D)$. If the trees are allowed to grow until each leaf contains a single observation, as done in our Golem implementation, the number of tiles M corresponds to the number of observations K in the dataset $\mathcal{D}_K$. Typically, these are the observations for which one would like to re-evaluate the merits. In addition, we expect the number of trees T and the input dimensionality D to generally be small with respect to the number of observations. Hence, in a typical asymptotic scenario we have that $M = S \gg T, D$ with Golem displaying a quadratic runtime $\mathcal{O}(n^2)$ that depends on the number of observations collected. The time complexity can, however, be further reduced to $\mathcal{O}(S)$ by defining a maximum tree depth that would bound M. Figure S2 shows Golem's run time when varying S, T, M, and D as discussed above. Note that, despite the quadratic scaling of the implementation, the run time has a small prefactor. In practice, robust merits for thousands of samples can be estimated on a single CPU core in a matter of seconds. For instance, evaluating the robust merits of 2500 samples using a surrogate model with 10 trees, for a two-dimensional problem, takes approximately 7 seconds on a single core of a 1.4 GHz Quad-Core Intel Core i5-8257U processor.

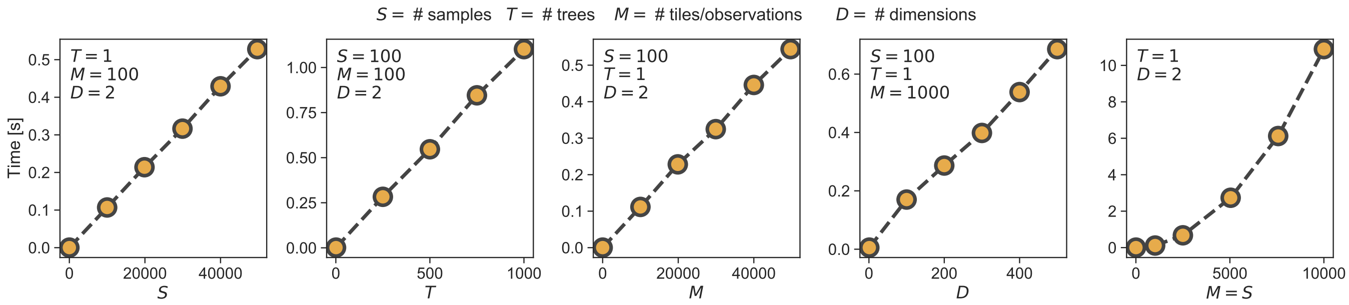

FIG. S2. Computational scaling of GOLEM with respect to the number of predicted samples (S), number of trees used as part of the surrogate model (T), number of leaves in each tree, which, in our implementation correspond to the number observations in the dataset used for training (M), and dimensionality of the optimization task (D).

S.2. SYNTHETIC BENCHMARKS

In the following, information regarding the synthetic benchmark functions used to evaluate GOLEM's performance, additional analyses, and the details of all results obtained, are provided.

A. Benchmark functions

In this work, we use three objective functions, which, given different assumed input uncertainties, create the different robust objective functions used as synthetic benchmarks. We refer to these three objective functions as *Bertsimas*, *Cliff*, and *Sine*. The *Bertsimas* function is taken from previous work on robust optimization by Bertsimas *et al.*[5]. It corresponds to the following nonconvex polynomial function for $x \in [-1, 3.2]$ and $y \in [-0.5, 4.4]$:

$$\begin{aligned} f(x,y) = {} & 2x^6 - 12.2x^5 + 21.2x^4 + 6.2x - 6.4x^3 - 4.7x^2 + y^6 - 11y^5 + 43.3y^4 - 10y - 74.8y^3 \\ & + 56.9y^2 - 4.1xy - 0.1y^2x^2 + 0.4y^2x + 0.4x^2y. \end{aligned} \tag{31}$$

In this work we place an upper bound to the function codomain, such that the *Bertsimas* function used in practice is $\min(f(x,y), 80)$. This was done to avoid the extremely large values present outside its optimization domain. The *Cliff* function is introduced in this work and is defined as follows, with $\boldsymbol{x} \in [0, 5]^D$, where D is the number of dimensions:

$$f(\boldsymbol{x}) = \sum_{d=1}^{D} \frac{10}{1 + 0.3e^{6x_d}} + 0.2x_d^2. \tag{32}$$

The *Sine* function is also introduced in this work and is defined as follows, with $\boldsymbol{x} \in [-1, 1]^D$:

$$f(\boldsymbol{x}) = \sum_{d=1}^{D} \sin(2\pi x_d^2) + x_d^2 + 0.2x_d. \tag{33}$$

The global minimum of the *Bertsimas* function is at $(x^*, y^*) = (2.8, 4.0)$, the minimum of *Cliff* is at $\boldsymbol{x}^* = (1.02874)^D$, and that of *Sine* is at $\boldsymbol{x}^* = (-0.85297)^D$.

Discrete versions of *Bertsimas* and *Cliff* were obtained by discretizing and scaling their domain onto a 22×22 grid, such that $x \in \mathbb{N} \mid 1 \leq x \leq 22$ and $y \in \mathbb{N} \mid 1 \leq y \leq 22$. In these cases, the gobal minima are found at $(x^*, y^*) = (20, 20)$ and $(x^*, y^*) = (5, 5)$ for the *Discrete Bertsimas* and *Discrete Cliff* functions, respectively.

The robust objective functions S1–S8 are obtained by transforming the above functions based on specific input distributions, as shown in Figure 3 and detailed in Table S1. For continuous functions, we used the *Normal*, *Gamma*, and *Uniform* distributions with various scales. For discrete functions, we used the *Poisson* and *Discrete Laplace*[6] distributions. However, note that any parametric distribution can in principle be used to model input uncertainty. In our GOLEM package, we implemented a Gamma distribution parametrized by its standard deviation and with variable

lower or upper bounds. Similarly, we allow shifting the Poisson distribution such that any lower bound can be chosen. Details of these implementations can be found in Golem's GitHub repository[7].

For all objective functions in Table S1, a close numerical approximation of their corresponding robust objective was obtained with Golem using a dense grid of 40,000 samples. These samples extended beyond the optimization domain of each objective function, to be able to accurately model the objective function across all accessible regions of input space. For surfaces associated with unbounded probability distributions, samples were taken up to two standard deviations away from the optimization domain boundaries.

Label	Function	Optimization domain	Probability distribution	Scale†	Support	Improvement‡
S1	Cliff	$x_i \in [0, 5]$	Normal	1.0	$x_i \in \mathbb{R}$	25%
S2	Cliff	$x_i \in [0, 5]$	Gamma	2.0	$x_i \in (-\infty, 5]$	51%
S3	Bertsimas	$x_0 \in [-1, 3.2]$	Uniform	1.5	$x_0 \in [-1.75, 3.95]$	34%
		$x_1 \in [-0.5, 4.4]$			$x_1 \in [-1.25, 5.15]$	
S4	Bertsimas	$x_0 \in [-1, 3.2]$	Normal	0.8	$x_i \in \mathbb{R}$	53%
		$x_1 \in [-0.5, 4.4]$				
S5	Sine	$x_i \in [-1, 1]$	Uniform	0.5	$x_i \in [-1.25, 1.25]$	26%
S6	Sine	$x_i \in [-1, 1]$	Normal	0.2	$x_i \in \mathbb{R}$	26%
S7	Discrete Cliff	$x_i \in \mathbb{N} \mid 1 \leq x \leq 22$	Discrete Laplace	3	$x_i \in \mathbb{N}$	18%
S8	Discrete Bertsimas	$x_i \in \mathbb{N} \mid 1 \leq x \leq 22$	Poisson	n.a.	$x_i \in \mathbb{N} \mid x \geq 1$	74%

TABLE S1. Details of the synthetic benchmark functions used to evaluate Golem. †One standard deviation for Normal, Gamma, and Discrete Laplace distributions; range for Uniform distributions; not applicable to Poisson distributions as not parametrized by scale. ‡ Measure of the improvement in robustness between the minimum of the objective function and that of the robust objective function, relative to the range of the co-domain of the robust objective function.

B. Bias due to boundary effects

When the input parameters are noisy, the realized location of the queries does not correspond to that of the requested location. As a consequence, while one requests only locations within the bounds of the defined optimization domain, objective function evaluations outside of these bounds are possible. To know the true robustness of each solution within the optimization domain, one would thus need to know how the objective function behaves outside of the bounds of the optimization. As we approximate the objective function with a machine learning model, based on a dataset that has no samples outside the optimization domain, the surrogate model built is likely to be poor far outside the boundaries of the optimization domain. This lack of information results in a biased robust surrogate model also in the limit of infinite sampling within the optimization domain. This effect is exemplified by Figure S3, in which a surrogate robust objective was built with Golem using a dataset containing 10,000 samples equally spaced within the optimization domain only. Another consequence of this boundary effect is that Golem's estimates of the robust objective tend to be less accurate for points close to the boundaries of the optimization domain (Figure S4).

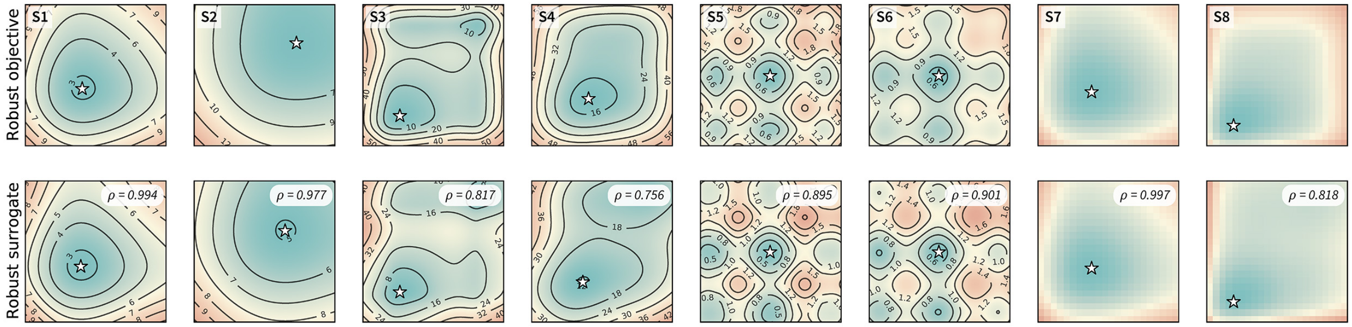

FIG. S3. Converged robust surrogate estimates. The first row shows the true robust objective functions, which depend on the behavior of the objective function also outside the optimization domain shown. The second row shows converged robust surrogate estimates based on a regular grid of 10,000 points within the optimization domain. Spearman's correlation (ρ) between the robust objective and its surrogate model are shown for each benchmark surface. Deviations from the ideal $\rho = 1$ correlation are due to the inability of Golem's surrogate model (in this case, a single regression tree) to accurately capture the objective function's behavior beyond the optimization domain due to a lack of data in those regions.

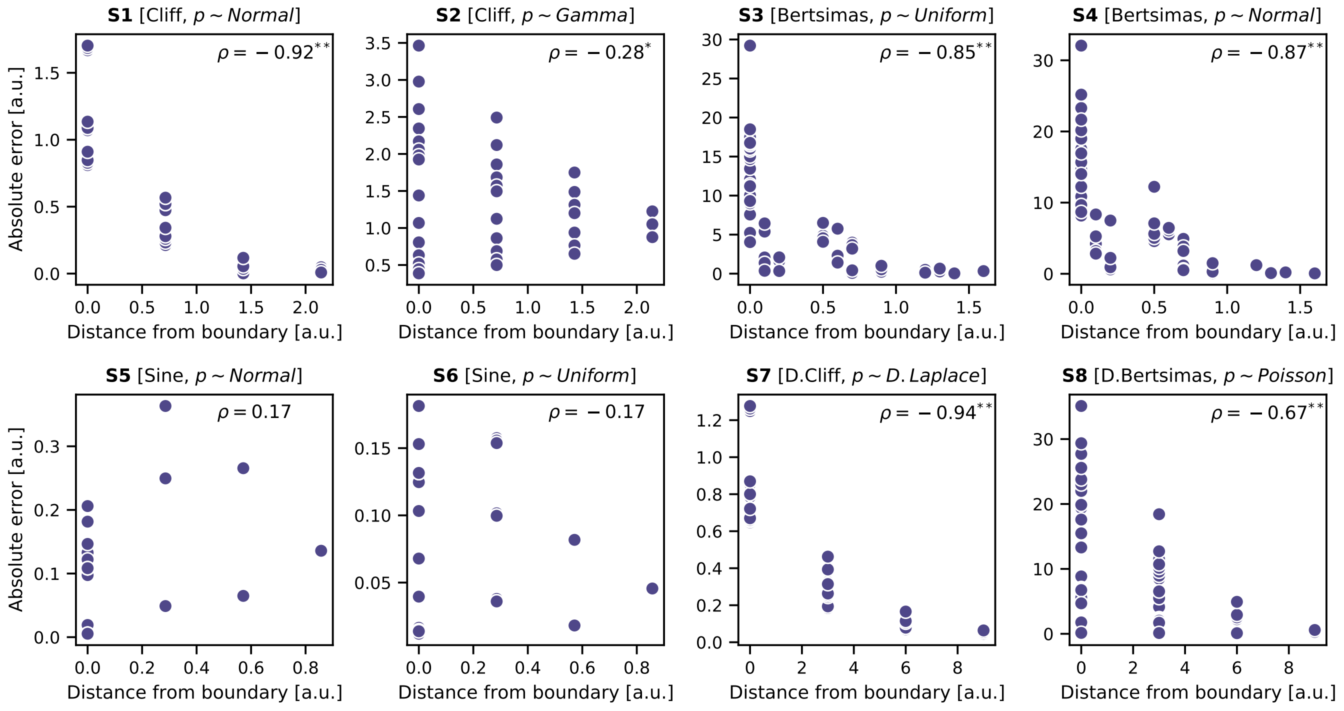

FIG. S4. Relationship between distance from optimization boundaries and robustness estimate error. The data shown are for 64 points uniformly sampled on a grid, as shown in the fourth row of Figure 3. The errors are the difference between the robustness estimates obtained with GOLEM based on 64 datapoints and the ground truth estimate obtained as described in section S.2.A. On each plot we report the Spearman's correlation (ρ) between the errors and distances. Correlations with p-values less than 0.05 are marked with one star, and those less than 0.01 are marked with two. In the majority (six out of eight) of the test surfaces considered, there is a significant negative correlation between boundary distance and absolute errors.

C. Cumulative robust regret as performance measure

To compare the relative optimization performance of all algorithms tested on specific benchmark functions $f(\cdot)$ we used the following definition of cumulative robust regret:

$$\sum_{k=1}^{K} g\Big(\operatorname*{argmin}_{\boldsymbol{x} \in \boldsymbol{x}_{1:k}} \hat{g}(\boldsymbol{x}_{1:k}) \Big), \tag{34}$$

where $\boldsymbol{x}_{1:k}$ are all samples collected up to iteration k, $\hat{g}(\cdot)$ is the estimate of the robust merits obtained with GOLEM after training on $\mathcal{D}_{1:k}$, and $g(\cdot)$ is the true robust objective function. The true robust objective is obtained as an accurate GOLEM estimate by using a dense grid of $40,000$ samples, as mentioned in section S.2.A. To measure the performance of each optimization algorithm without GOLEM as the baseline, the original values of the merits, as obtained from the objective function $f(\cdot)$, were used instead of those derived with $\hat{g}(\cdot)$. Effectively, at each iteration, after having collected one additional sample, we estimate which one (among all samples collected thus far) has the best robust merit as estimated by GOLEM. Then, we take the true robust merit of this sample. All true robust merits obtained in this way for $k = 1$ to $k = K$, where K is the total number of samples, are then summed. This measure quantifies the speed at which the optimization algorithm has discovered better robust solutions. Given we are performing minimizations, the lower the cumulative regret, the better performing the algorithm is. For visualization and interpretation purposes, we normalize the values of cumulative regrets obtained in this way for each test on a specific benchmark surface. Hence, within each plot (e.g., in Figure 4), a value of zero corresponds to the best cumulative regret observed for that surface, and a value of one to the worst. Note that, because this measure is not normalized across benchmark surfaces, comparisons are meaningful only with respect to a specific surface.

D. Impact of uniformity and sampling of the boundaries

In the optimization benchmarks carried out we noted that *Grid* generally performed better than *Random*. We hypothesized this might be caused by one or two features of these approaches. First, the difference in performance could be due the more uniform sampling of input space that is guaranteed with *Grid*. To test this hypothesis, we performed optimizations, in the noiseless setting, with a Sobol sequence (we refer to this approach as *Sobol*), which samples input space more uniformly than *Random* but less than *Grid*. Second, the performance difference could be due to the fact *Grid* guarantees good sampling of the boundaries of the optimization domain. As discussed in section S.2.B, boundaries effect are present due to the lack of information on the objective function's behavior outside the optimization domain. To test this hypothesis, we benchmarked two additional approaches, in which we augmented *Random* and *Sobol* with samples at exactly the optimization domain boundaries. We placed these samples at the same locations of those in *Grid*. We refer to these two approaches as *Sobol-Edge* and *Random-Edge*. In all cases, we allowed 196 objective function evaluations in total, as in the benchmarks described in section V. Figure S5 summarizes the results obtained with the above approaches against benchmark functions S1–S6. We found that the unlikely sampling of the boundaries was the primary factor negatively impacting the performance of *Random* when used in conjunction with Golem. The sampling of the boundaries present in *Sobol-Edge* and *Random-Edge* allowed Golem to better estimate the robustness of the input parameters collected, resulting in better performance for these two approaches as compared to *Sobol* and *Random*. The effect of uniform sampling was not noticeable in S1–S4, with *Grid*, *Sobol-Edge*, and *Random-Edge* performing equally well. A small difference in performance was noticeable only for the rougher surfaces S5 and S6. There, *Grid* performed better than *Sobol-Edge*, which in turn was better than *Random-Edge*, as expected if sampling uniformity were beneficial to robust optimizations with Golem.

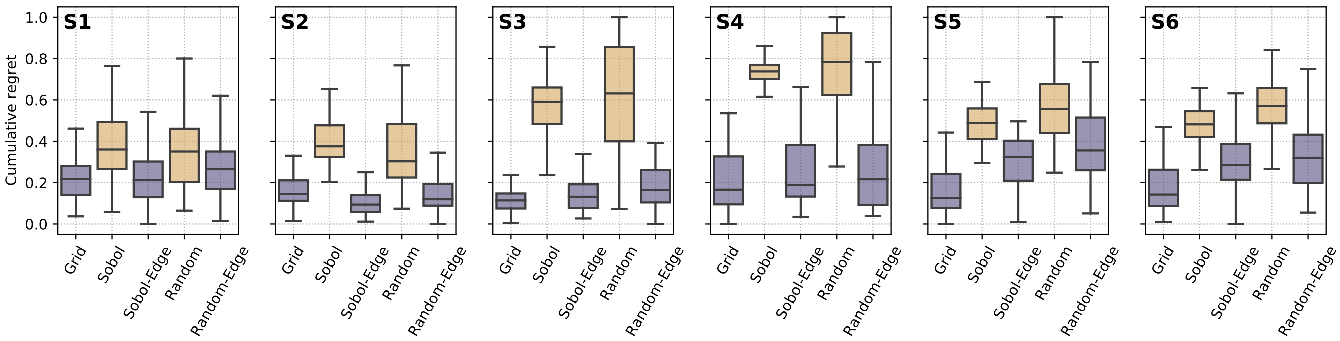

FIG. S5. Optimization performance of different design of experiment approaches when used with Golem. Box plots show the distributions of cumulative regrets obtained across 50 optimization repeats. The boxes show the first, second, and third quartiles of the data, with whiskers extending up to 1.5 times the interquartile range. Boxes for approaches that sampled the boundaries of the optimization domain are shown in purple, while those for approaches that did not sample boundaries exactly are shown in yellow.

E. Influence of approximate surrogate model

As discussed in section V.B, when queries are noisy, building an accurate surrogate model is challenging because the objective function is not evaluated at the desired queried locations. As a consequence, the data $\widetilde{\mathcal{D}}_K = \{\boldsymbol{x}_k, \widetilde{f}_k\}_{k=1}^{K}$ available to train the surrogate model is mismatched. However, while Golem does not take into account input noise at training time, it does so at the inference stage when estimating robustness (i.e., $g(\boldsymbol{x})$). Golem may be seen as trying to estimate where the value of $\widetilde{f}_k$ for the query $\boldsymbol{x}_k$ might have come from. Because of this, Golem is able to recover reasonably accurate estimates of $g(\boldsymbol{x})$ even when the correlation between true, f_k, and observed, $\widetilde{f}_k$, objective function values is lost. This effect is shown in Figure S6. The first row shows the true robust objective functions that we introduced in Figure 3, while the other rows show three Golem estimates based on a dataset $\widetilde{\mathcal{D}}_K$ comprised of 64 datapoint collected under severe input uncertainty (as defined in Table S1). As a consequence, the correlation between true and observed objective function values, for the chosen queries locations is low, and in some cases effectively lost. However, Golem manages to recover moderate $(0.4 - 0.7)$ to strong $(0.7 - 0.9)$ correlations between its robustness estimates and the true robust objective values.

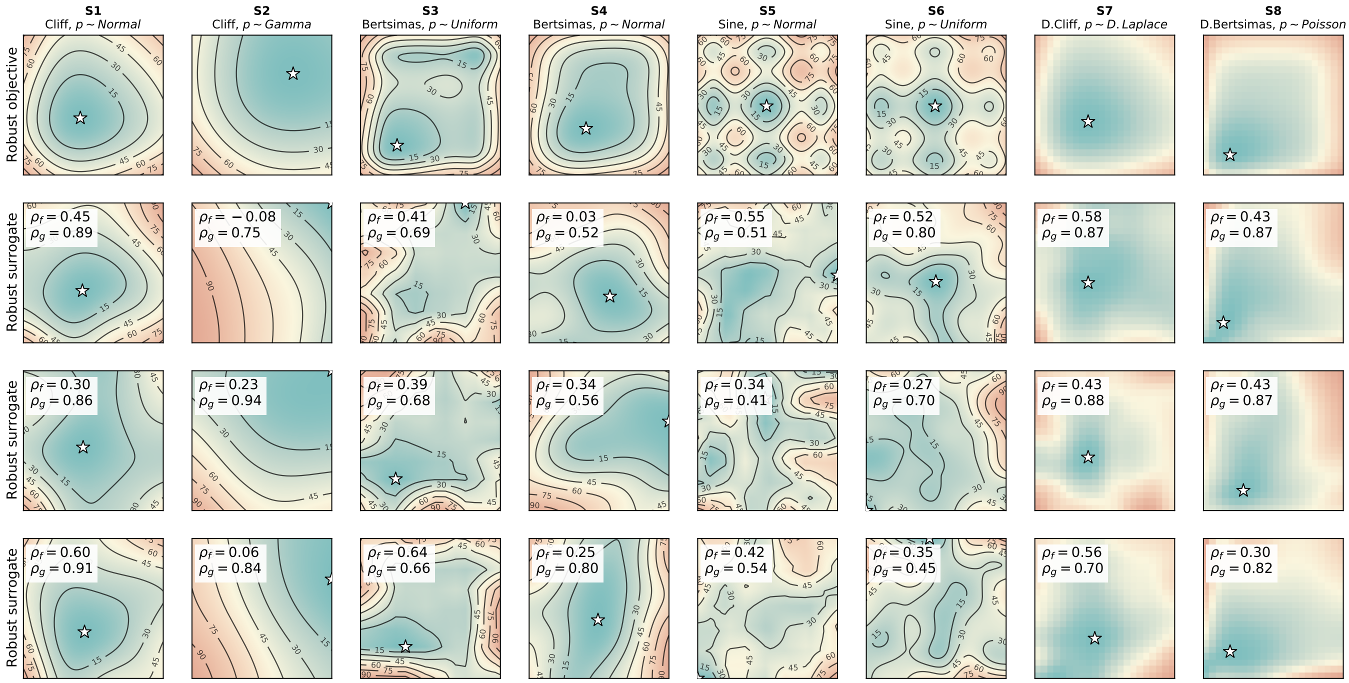

FIG. S6. Golem's robustness estimates based on noisy data. The first row shows the true robust objective functions. The other rows show Golem estimates based on three different noisy datasets $\widetilde{\mathcal{D}}_K$. In all cases, 64 datapoints were sampled under severe input noise, according to the uncertainties defined in Table S1. On each plot, the Spearman's correlation between true and observed objective function values for these 64 datapoints is reported as ρ_f. The correlation between Golem's robustness estimates (based on these noisy datapoints) and the true robust objective values is reported as ρ_g. In the vast majority of cases, ρ_g was much larger than ρ_f, which means that Golem was able to recover correlations with the robust objective despite having to rely on poorly informative samples of the objective function. The most striking example was observed for surface S2, where in one case there was a negative correlation between the true and sampled objective function values ($\rho_f = -0.08$), while Golem's predictions showed a strong positive correlation ($\rho_g = 0.75$).

F. Influence of the type and size of tree ensemble

Golem can be used with several tree ensemble algorithms. We tested the performance of Golem where the surrogate function is modeled with regression trees, random forest[3], and extremely randomized trees[4]. The *scikit-learn*[8] implementations of these algorithms were used (`DecisionTreeRegressor`, `RandomForestRegressor`, and `ExtraTreesRegressor`, respectively). In addition to testing the performance of a single regression tree, we also fitted ensembles of 10, 20, and 50 trees for all above-mentioned algorithms. Note that the regression tree algorithm used is not fully deterministic, such that different trees in the ensemble can correspond to different surrogate models. While the input dataset is not bootstrapped (like in random forest)[3] and thresholds for splitting nodes are not chosen at random (like in extremely randomized trees)[4], multiple splits can provide the same mean-square-error improvement, and a specific split is then chosen at random among these.

Figures S7 and S8 provide a summary of Golem's relative performance when using (i) different tree-based machine learning models (regression trees, random forest, and extremely randomized trees), and (ii) ensemble of trees of different sizes (1, 10, 20, 50). Figures S7 shows the normalized cumulative regret values (section S.2.C) for the results obtained with the six optimization algorithms tested, on the eight benchmark functions employed, and in the noiseless query setting. Figures S8 shows the same results, but for optimizations in the noisy query setting. Note that, because of the normalization of the cumulative regrets, results can be compared only within, and not across, subplots.

Figures S9 and S10 provide a summary of Golem's relative performance when using different tree-based models of different size, on high-dimensional benchmark surfaces. All these surfaces are higher-dimensional versions of the surface S1 (from three to six dimensions), where the first two dimensions are uncertain, while the additional ones are always considered to be noiseless. In these high-dimensional tests, it is possible to notice how surrogate models based on random forest and extremely randomized trees provided slightly better performance than regression trees.

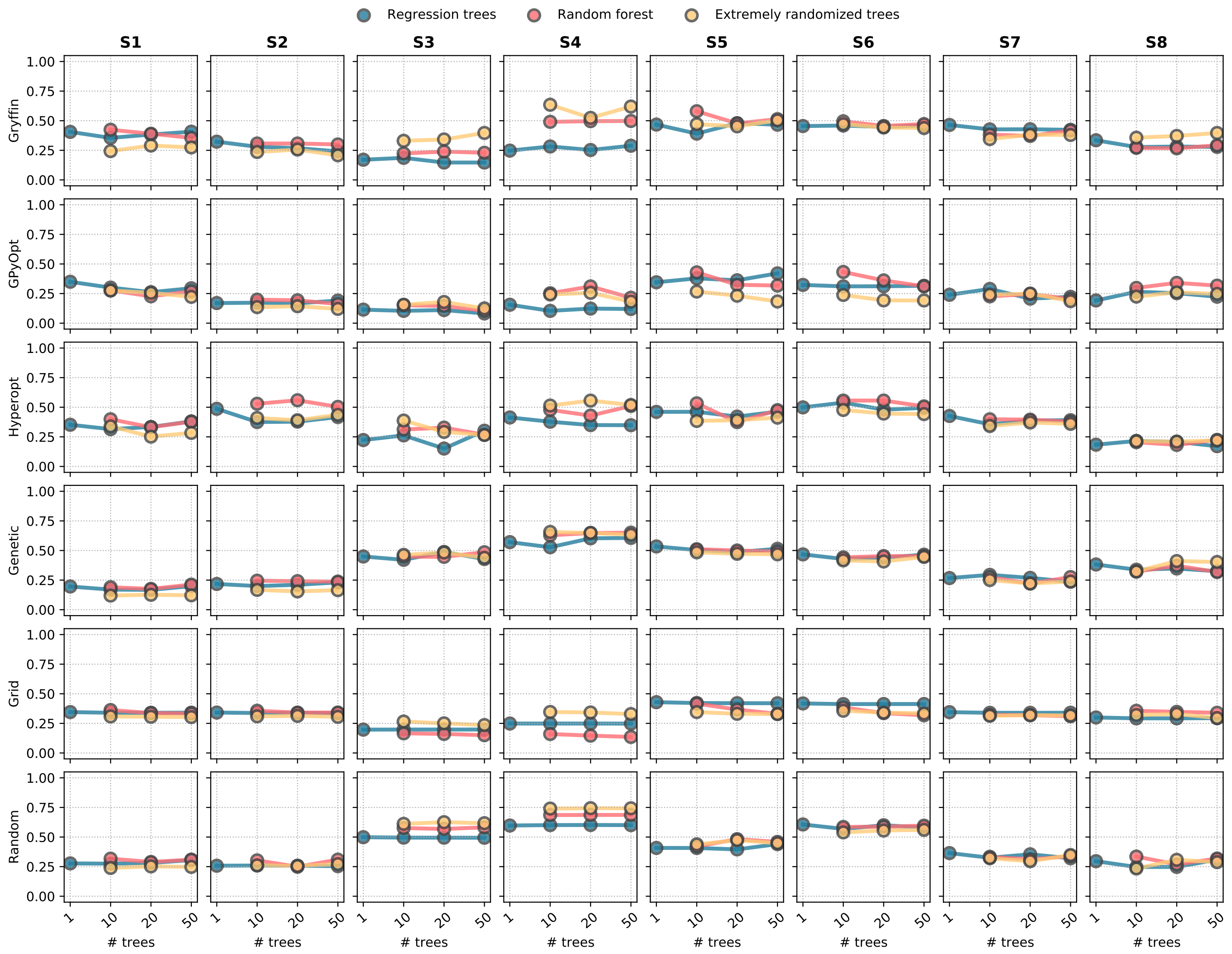

FIG. S7. Influence of the type and size of tree ensemble on Golem's performance for optimizations in the noiseless query setting. Shown are cumulative regret values, normalized within each subplot, and averaged across 50 repeated optimization runs. Each subplot refers to optimizations performed on a different benchmark surface and with a different algorithm in conjunction with Golem.

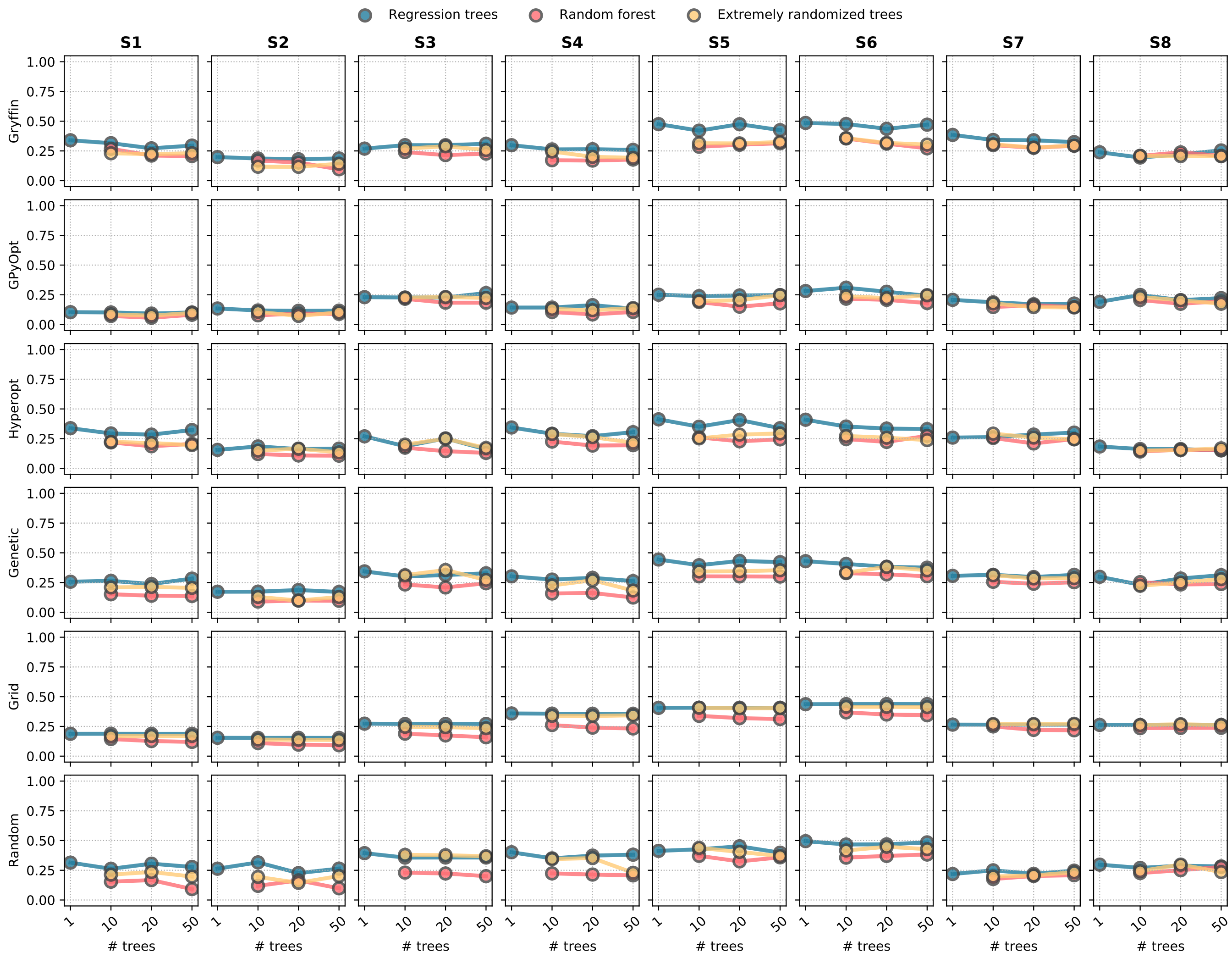

FIG. S8. Influence of the type and size of tree ensemble on Golem's performance for optimizations in the noisy query setting. Shown are cumulative regret values, normalized within each subplot, and averaged across 50 repeated optimization runs. Each subplot refers to optimizations performed on a different benchmark surface and with a different algorithm in conjunction with Golem.

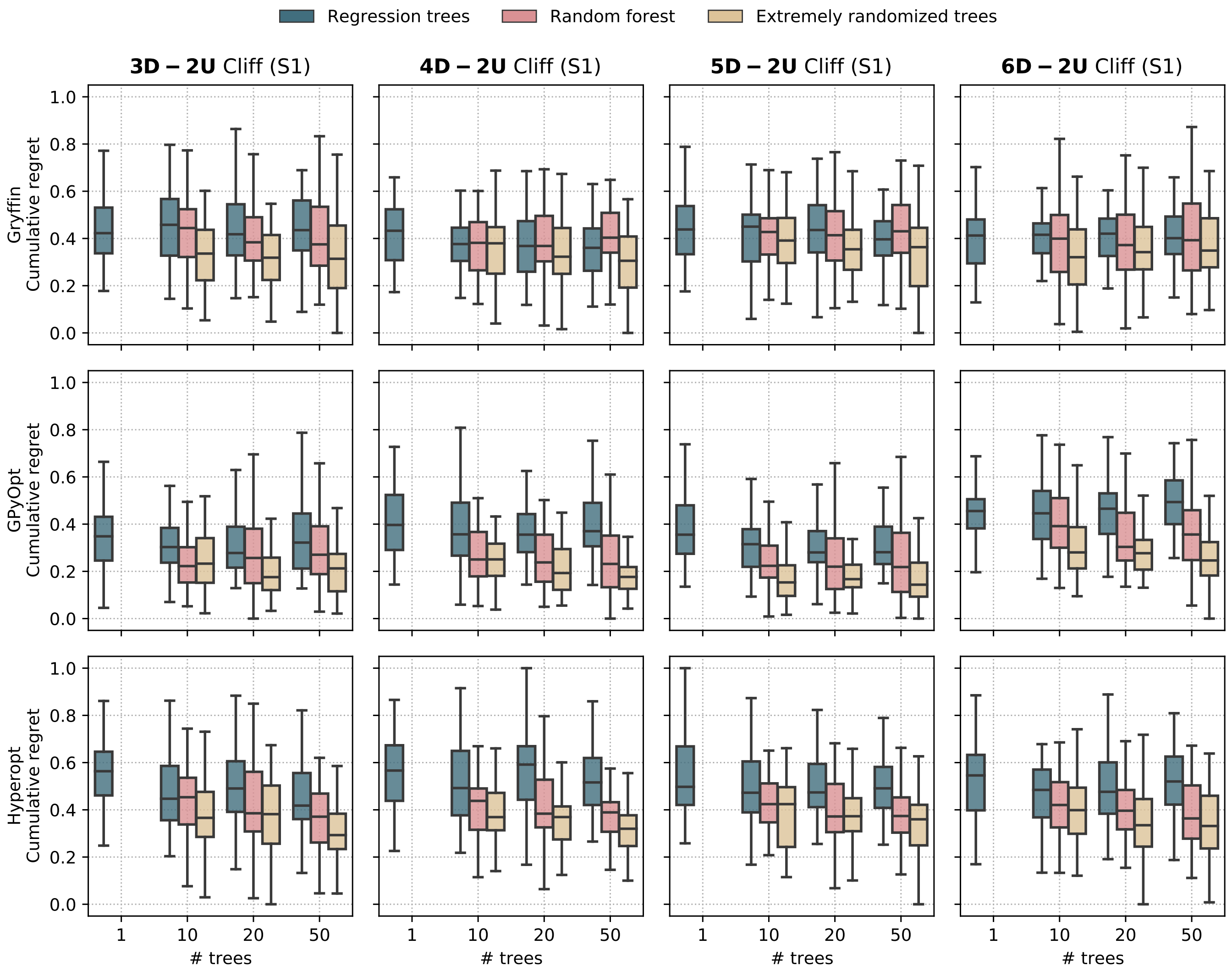

FIG. S9. Influence of the type and size of tree ensemble on Golem's performance for high-dimensional optimizations in the noiseless query setting. The distributions of cumulative regret values, normalized within each subplot, across 50 repeated optimization runs are shown. Each subplot refers to optimizations performed on surfaces of increasing dimensionality and with a different algorithm.

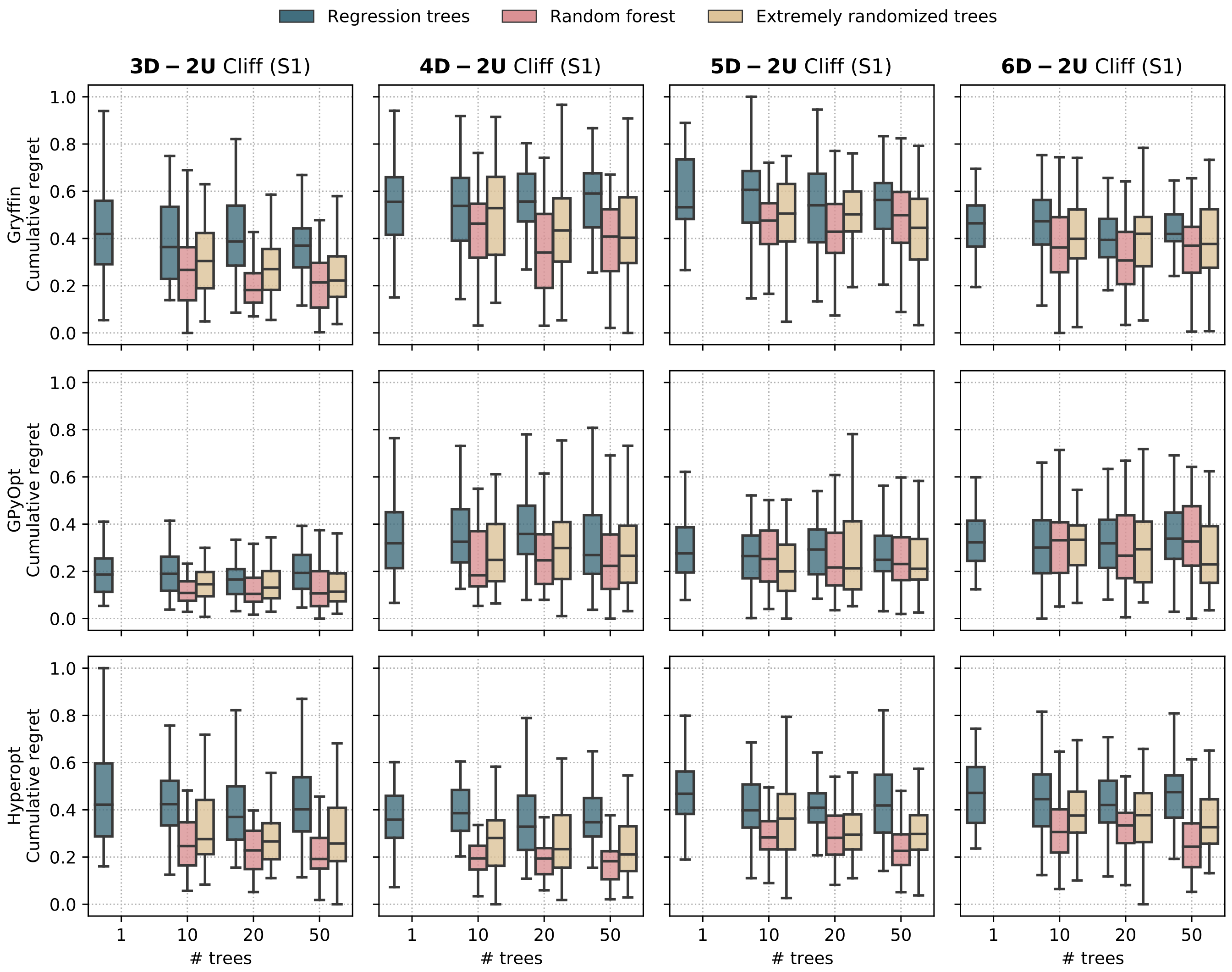

FIG. S10. Influence of the type and size of tree ensemble on GOLEM's performance for high-dimensional optimizations in the noisy query setting. The distributions of cumulative regret values, normalized within each subplot, across 50 repeated optimization runs are shown. Each subplot refers to optimizations performed on surfaces of increasing dimensionality and with a different algorithm.

G. Influence of the number of uncertain variables

The larger the dimensionality of the problem, and the larger the number of uncertain input variables, the more challenging the robust optimization task is. We tested how GOLEM's performance is affected by the presence of additional noise-free and noisy input variables. All surfaces used in these tests are higher-dimensional versions of the surface S1 (from three to six dimensions), where between one and all of the available input variables are noisy. Figures S11 and S12 show the normalized cumulative regrets obtained for optimizations in the noiseless and noisy query setting, respectively. These results are discussed in V.C. In summary, we find that GOLEM is effective also on higher-dimensional surfaces. In fact, the benefits of using GOLEM become more marked the higher the number of uncertain inputs present in the optimization domain (i.e., the more uncertainty being present overall). On the other hand, given a fixed number of uncertain input variables, additional noiseless variables make it harder for GOLEM to enhance the performance of the optimization algorithm used. Importantly, GOLEM was almost never (one out of 108 tests) found to be detrimental to optimization performance.

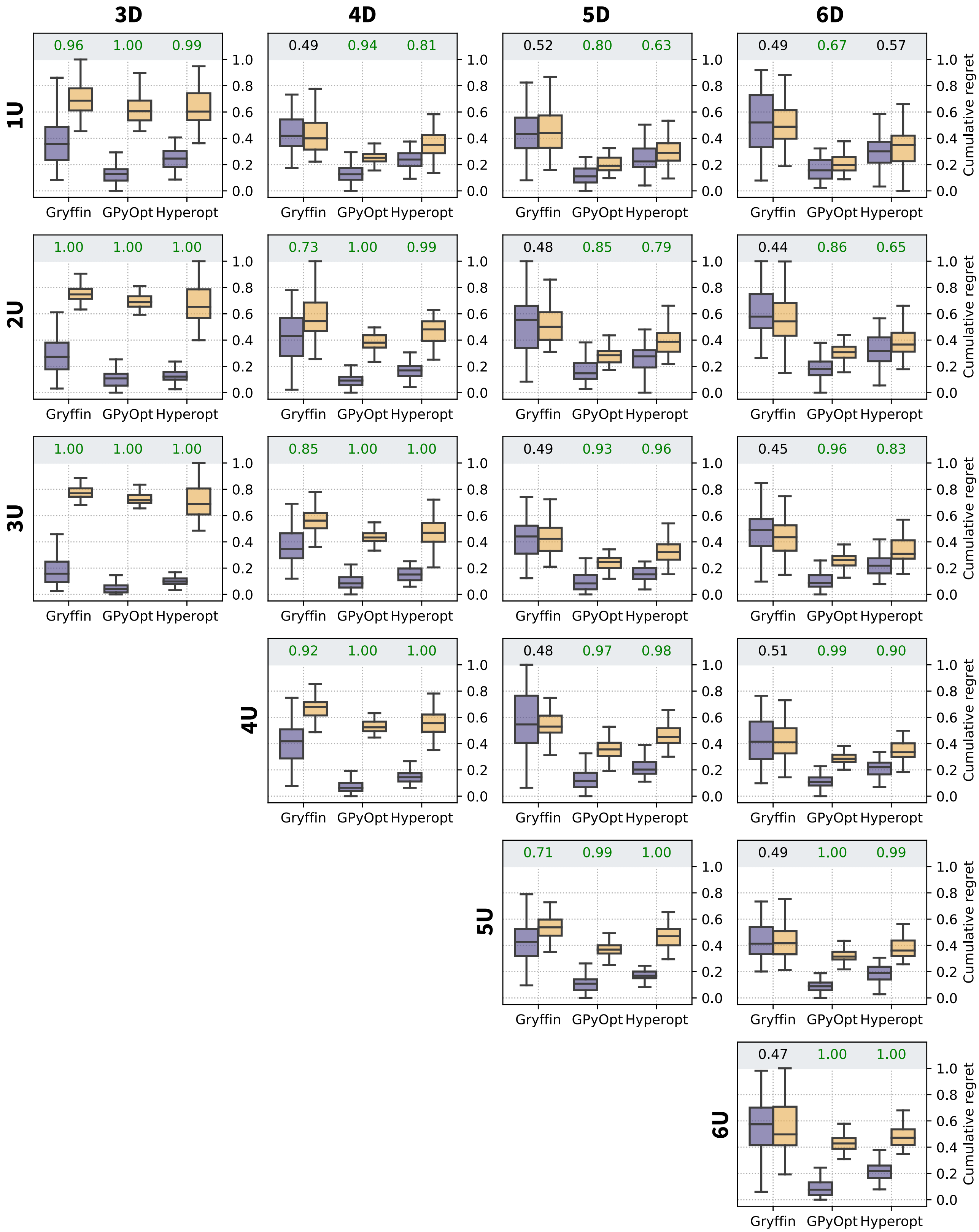

FIG. S11. Relative comparison of optimization performance obtained with and without GOLEM on the surface S1 with varying dimensions (3D−6D) and number of uncertain inputs (1U−6U) in the noiseless query setting. The regret distributions shown were obtained from optimizations that used GOLEM with an ensemble of 50 extremely randomized trees as the surrogate model. The boxes show the first, second, and third quartiles of the data, with whiskers extending up to 1.5 times the interquartile range. Results obtained with GOLEM are shown in purple, and those obtained without GOLEM in yellow. The probability of obtaining better performance with GOLEM, with the algorithms tested, is reported above each box. Statistically significant results ($\alpha = 0.05$) are highlighted in green (significant improvement when using GOLEM) and red (significant deterioration when using GOLEM).

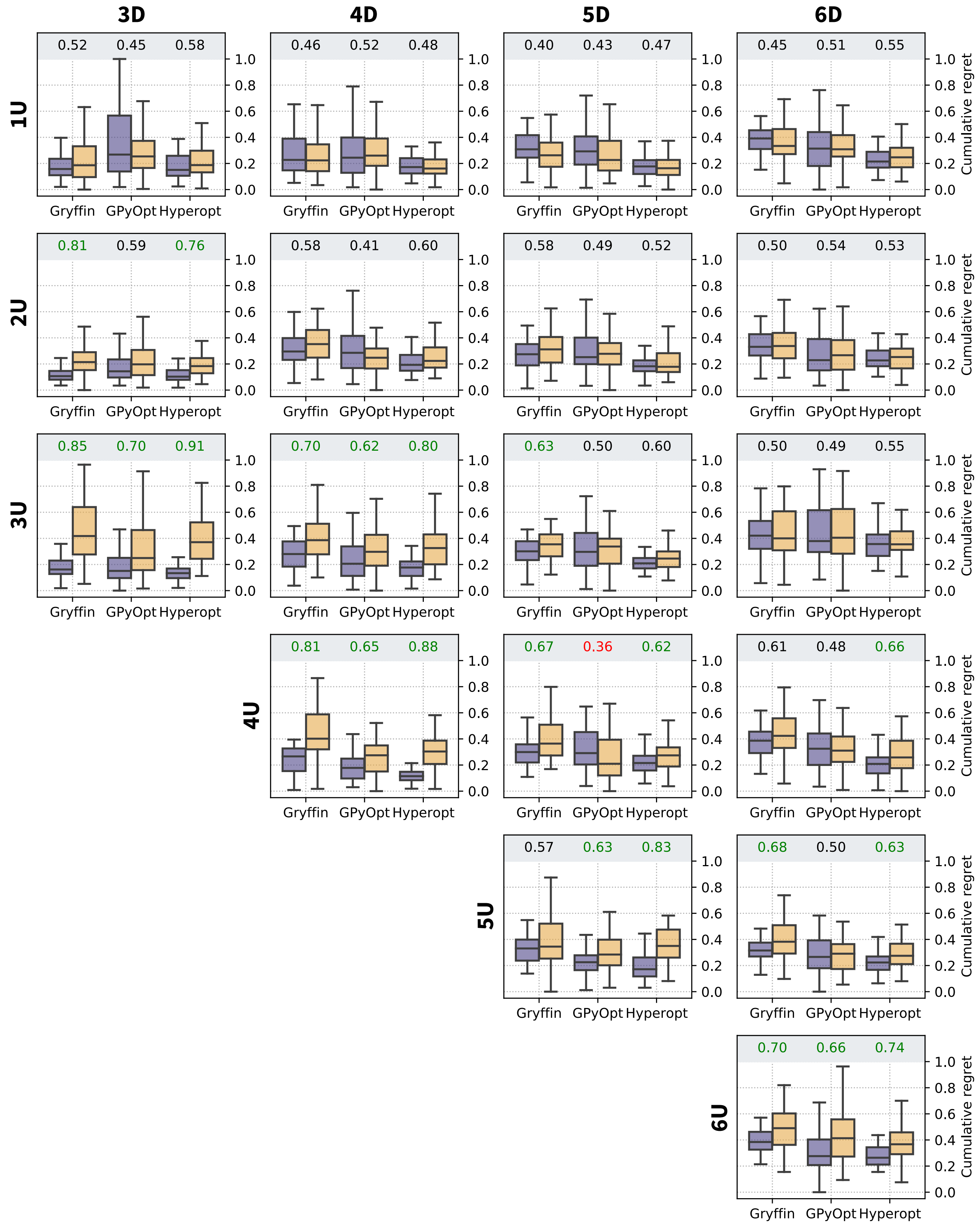

FIG. S12. Relative comparison of optimization performance obtained with and without Golem on the surface S1 with varying dimensions (3D−6D) and number of uncertain inputs (1U−6U) in the noisy query setting. The regret distributions shown were obtained from optimizations that used Golem with an ensemble of 50 extremely randomized trees as the surrogate model. The boxes show the first, second, and third quartiles of the data, with whiskers extending up to 1.5 times the interquartile range. Results obtained with Golem are shown in purple, and those obtained without Golem in yellow. The probability of obtaining better performance with Golem, with the algorithms tested, is reported above each box. Statistically significant results ($\alpha = 0.05$) are highlighted in green (significant improvement when using Golem) and red (significant deterioration when using Golem).

S.3. ANALYSIS AND OPTIMIZATION OF AN HPLC PROTOCOL

In this section we provide more details on the setup and results concerning the example application on the calibration of an HPLC protocol. In this example, the experimental HPLC response depends on six tunable parameters. These controllable input parameters (shown in Figure 7a) are the following: (P1) volume of the sample loop and internal volume of the 2-way 6-port valve; (P2) volume required to draw the sample to the 2-way 6-port valve; (P3) volume required to drive the sample plug from the sample loop, through the in-line mixer, and to the second valve; (P4) draw rate of the sample pump; (P5) push rate of the push pump; (P6) time waited after drawing sample and before switching the first selection valve (to allow for equilibration of cavitation bubbles in the sample line and syringe). As discussed in the main text, Golem may be used to retrospectively analyze the experimental results, or to optimize the protocol assuming no prior knowledge.

A. Interaction between input uncertainties and optimum location

The uncertainty present in one input parameter affects the robustness merit of the solutions across the whole search space. As such, uncertainty in one parameter might affect the optimal setting for other parameters too. Figure S13 shows such an example to visually clarify this statement. Based on a surrogate model built on 1386 HPLC experiments, we can use Golem to investigate how the response surface is affected by uncertainty in the parameters P1 and P3. For ease of visualization, Figure S13 shows surfaces only with respect to P1 and P3, with the other parameters fixed according to the best performing sample collected (P2 $\approx$ 0.03 mL, P4 $\approx$ 2.4 mL/min, P5 $\approx$ 107 Hz, P6 $\approx$ 6.2 s). We consider the presence of input uncertainty in P1 and P3 individually, and then in P1 and P3 together. In all cases, we assume a normally distributed uncertainty with standard deviation corresponding to 10% of the parameter range (i.e., 0.008 for P1 and 0.08 for P3). The distribution is furthermore truncated at zero to avoid non-physical values of P1 or P3. Assuming uncertainty only in P1, the location of the optimum is shifted to slightly higher values of P1 (Figure S13). However, when assuming uncertainty in P3, the location of the optimum is shifted considerably towards higher values of P1, while leaving almost unaffected the location with respect to P3, the uncertain parameter. This effect is due to objective function dropping slightly more steeply towards zero at low P3 values also when P1 is low, while having a slightly broader maxima in the P3 dimension for higher values of P1. The net result of this effect is that, when considering uncertainty in both P1 and P3, the location of the optimum is shifted primarily in P1, yet it is determined mainly by the uncertainty in P3. These types of interactions between variables are difficult to discover by simple visual inspection of the surrogate model, and is one of the tasks in which the use of Golem proves useful.

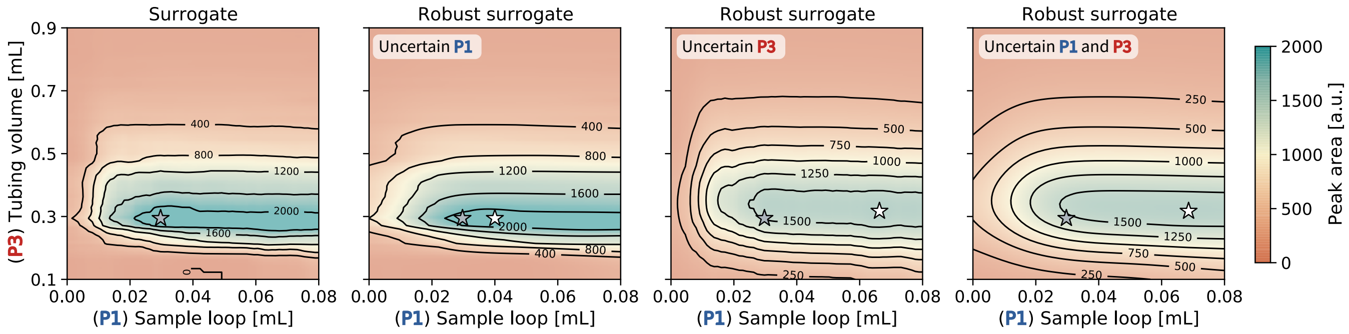

FIG. S13. Effect of uncertainty in P1 and P3 on the optimum location of the HPLC protocol. Golem's surrogate models are shown against the input parameters P1 and P3, while the other parameters are fixed. The plot on the left-hand side shows Golem's surrogate model, while the other plots show the robust counterpart when assuming uncertainty in P1, P3, and P1 and P3. We assume a normally distributed uncertainty with standard deviation corresponding to 10% of the parameter range (0.008 for P1 and 0.08 for P3). The distribution is furthermore truncated at zero to avoid non-physical values of P1 or P3. The location of the non-robust optimum is indicated by a gray star (as found by the experimental sample collected with highest peak area), while the location of the robust optima is indicated by a white star (as computed with Golem). These results show how uncertainty in P3 results in a large shift in optimum location along P1.

B. Optimization of a noisy HPLC protocol

In this example application, we assumed the presence of noise in parameters P1 and P3 while attempting to optimize the HPLC sampling protocol. We assumed this noise to be normally distributed and truncated at zero, with standard deviation of 0.008 mL for P1, and 0.08 mL for P3. The HPLC experiments were simulated with Olympus[9], which emulates the experimental HPLC response based on its six tunable parameters via a Bayesian Neural Network. The goal of the optimization was to achieve a protocol returning an expected peak area, $\mathbb{E}[Area]$, of at least 1000 a.u. As a secondary objective, we wanted to minimize the output variability, $\sigma[Area]$, as much as possible, as long as $\mathbb{E}[Area] > 1000$ a.u. Golem was used to estimate both the $\mathbb{E}[Area]$ and $\sigma[Area]$ during the optimization (Figure 8a), using 200 extremely randomized trees[4] as the surrogate model. The *Chimera*[10] scalarizing function was used to create a robust, multi-objective function to be optimized.

Similar to what we did to obtain a ground truth for the robust objectives for the analytical surfaces (Section S.2.A), a close numerical approximation of $\mathbb{E}[Area]$ and $\sigma[Area]$ was obtained by using a dense grid of uniformly distributed samples across the optimization domain. In this case, we sampled $8^6 = 262,144$ points from the Olympus experiment emulator to build a reference Golem model. These samples were extended beyond the optimization domain in P1 and P3 by two standard deviations. The approximate location of true robust optimum (Figure S14) was found with *Hyperopt* by optimizing the true robust, multi-objective function directly over 1000 iterations.

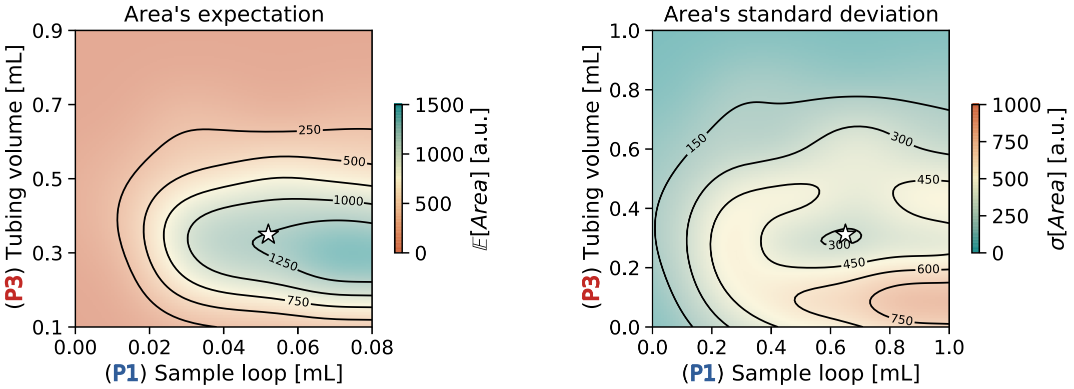

FIG. S14. Location of the true robust optimum identified and behavior of $\mathbb{E}[Area]$ and $\sigma[Area]$ around this optimum. The location of the global optimum is marked by a white star. It is located at P1 $\approx$ 0.052 mL, P2 $\approx$ 0.012 mL, P3 $\approx$ 0.35 mL, P4 $\approx$ 2.20 mL/min, P5 $\approx$ 84 Hz, P6 $\approx$ 5.9 s, where $\mathbb{E}[Area] = 1256$ and $\sigma[Area] = 288$.

[1] Hans Georg Beyer and Bernhard Sendhoff. Robust optimization - A comprehensive survey. *Computer Methods in Applied Mechanics and Engineering*, 196(33-34):3190–3218, 2007.

[2] Justin J Beland and Prasanth B Nair. Bayesian Optimization Under Uncertainty. *31st Conference on Neural Information Processing Systems (NIPS 2017) Workshop on Bayesian optimization (BayesOpt 2017)*, (1):1–5, 2017.

[3] Leo Breiman. Random Forests. *Machine Learning*, 45(1):5–32, 2001.

[4] Pierre Geurts, Damien Ernst, and Louis Wehenkel. Extremely randomized trees. *Machine Learning*, 63(1):3–42, 2006.

[5] Dimitris Bertsimas, Omid Nohadani, and Kwong Meng Teo. Robust Optimization for Unconstrained Simulation-Based Problems. *Operations Research*, 58(1):161–178, 2009.

[6] Seidu Inusah and Tomasz J. Kozubowski. A discrete analogue of the laplace distribution. *Journal of Statistical Planning and Inference*, 136(3):1090 – 1102, 2006.

[7] M. Aldeghi, F. Häse, R.J. Hickman, I. Tamblyn, and A. Aspuru-Guzik. Golem: An algorithm for robust experiment and process optimization. *GitHub*, https://github.com/aspuru-guzik-group/golem, 2021.

[8] F. Pedregosa, G. Varoquaux, A. Gramfort, V. Michel, B. Thirion, O. Grisel, M. Blondel, P. Prettenhofer, R. Weiss, V. Dubourg, J. Vanderplas, A. Passos, D. Cournapeau, M. Brucher, M. Perrot, and E. Duchesnay. Scikit-learn: Machine learning in Python. *Journal of Machine Learning Research*, 12:2825–2830, 2011.

[9] Florian Häse, Matteo Aldeghi, Riley J. Hickman, Loïc M. Roch, Melodie Christensen, Elena Liles, Jason E. Hein, and Alán Aspuru-Guzik. Olympus: a benchmarking framework for noisy optimization and experiment planning, 2020.

[10] Florian Häse, Loïc M Roch, and Alán Aspuru-Guzik. Chimera: enabling hierarchy based multi-objective optimization for self-driving laboratories. *Chemical Science*, 9(39):7642–7655, 2018.

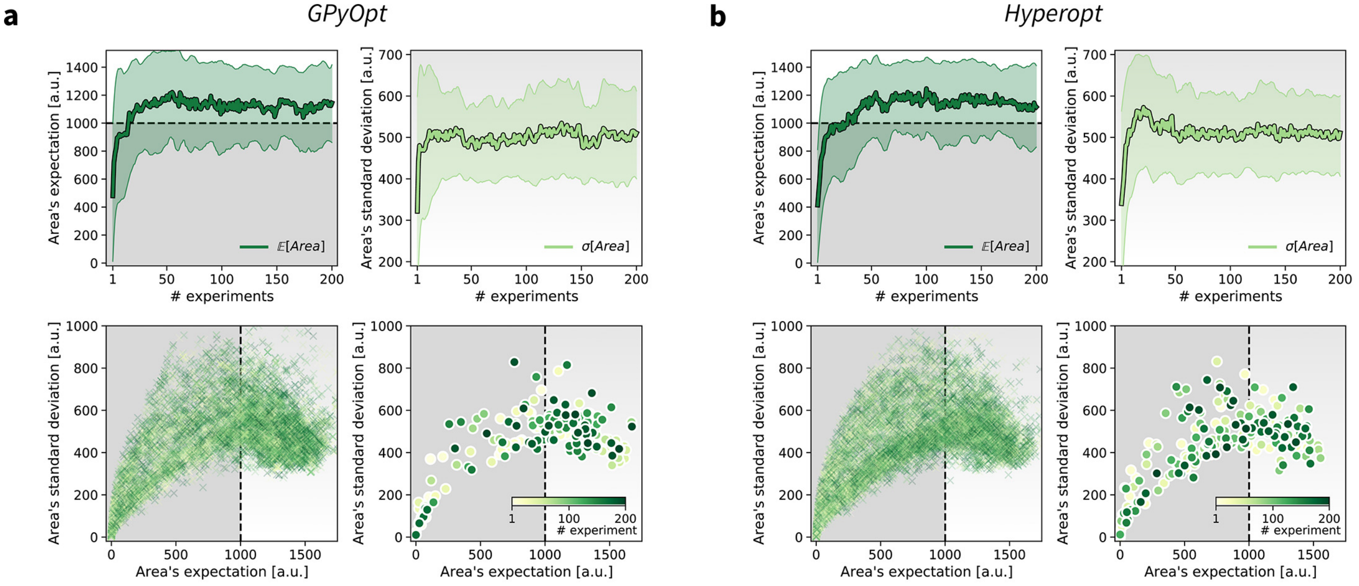

FIG. S15. Results of 50 optimization repeats performed with (a) *GPyOpt* and (b) *Hyperopt*. In both cases, optimization traces for the primary and secondary objectives are shown (average and standard deviation). All objective function values sampled during the optimization runs are shown in the bottom-left panels. Objective function values sampled during an example optimization run are shown in the bottom-right panels, with each experiment color-coded (yellow to dark green) to indicate at which stage of the optimization it was performed.

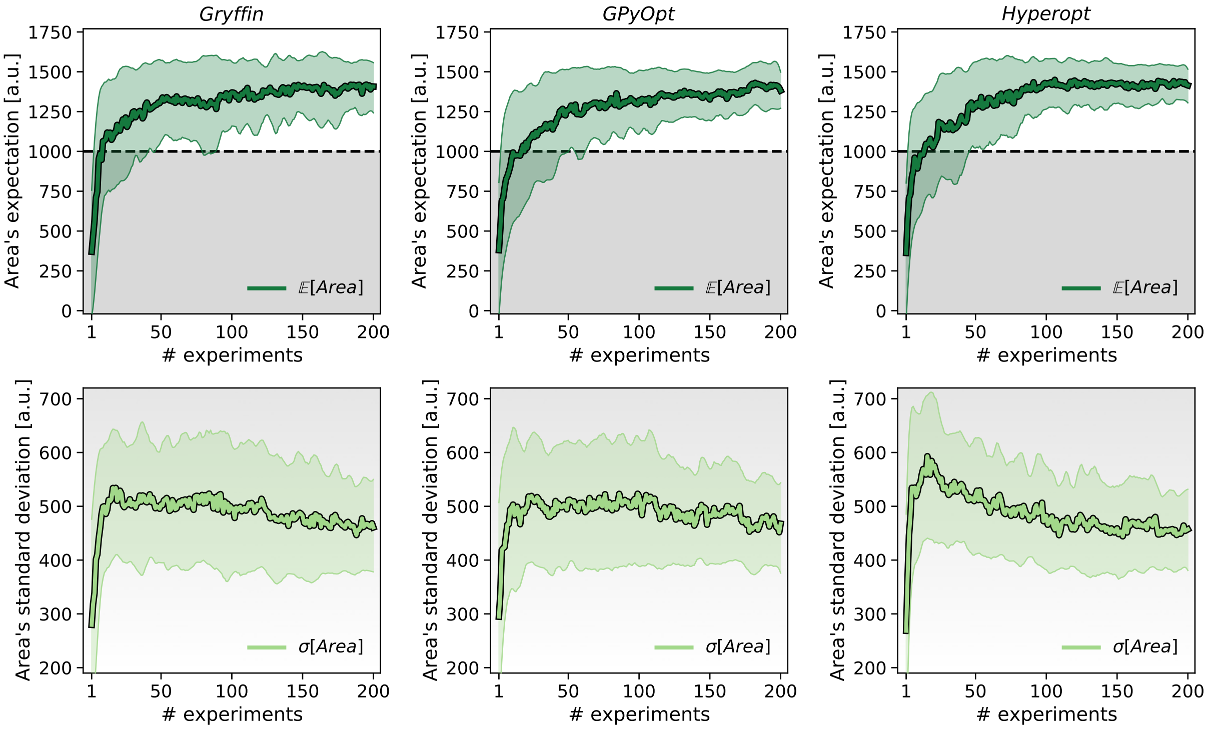

FIG. S16. Traces of 50 optimization repeats in which the primary objective was constrained to a lower-bound estimate of the peak area's expectation, $\mathbb{E}[Area] - 1.96 \times \sigma(\mathbb{E}[Area])$, corresponding to the lower bound of the 95% confidence interval of GOLEM's estimates. After 200 experiments, *Gryffin*, *GPyOpt*, and *Hyperopt* all correctly identified solutions with $\mathbb{E}[Area] > 1000$ a.u. in all 50 repeated optimization runs.

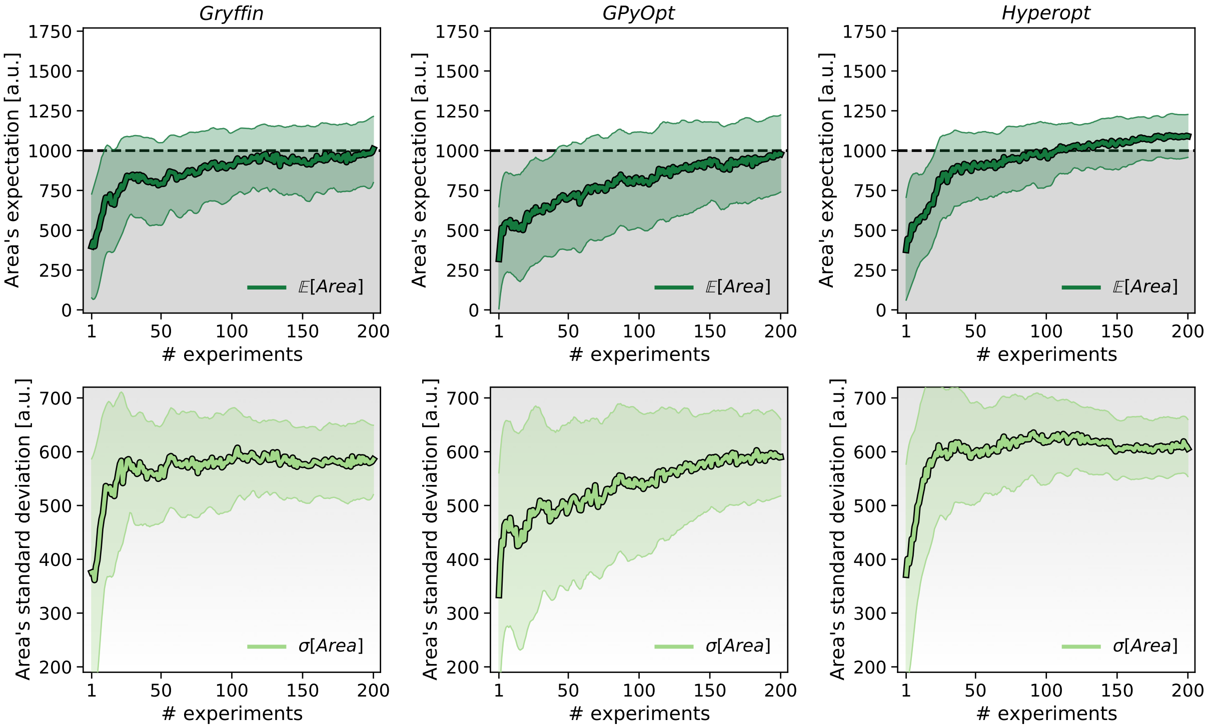

FIG. S17. Traces of 50 optimization repeats in which all six input parameters were noisy. The primary objective was constrained to a lower-bound estimate of the peak area's expectation, $\mathbb{E}[Area] - 1.96 \times \sigma(\mathbb{E}[Area])$, corresponding to the lower bound of the 95% confidence interval of Golem's estimates. With more overall noise in the input experimental conditions, the optimization takes longer than in the previous example with only two noisy variables. However, with Golem, all approaches still managed to optimize the peak area's expectation (i.e., $\mathbb{E}[Area]$ increases with more experiments performed and, on average, reaches the targeted value of 1000 a.u.). After 200 experiments, *Gryffin* correctly identified solutions with $\mathbb{E}[Area] > 1000$ a.u. in 42% of the optimization runs, *GPyOpt* in 70% of the optimization runs, and *Hyperopt* in 78%.
